# Exploring Compassionate Care Patterns Among Nurses and Its Associations With Resilience: Multilevel Contextual Effects Modeling

**DOI:** 10.1155/jonm/9518080

**Published:** 2025-02-17

**Authors:** Noushin Mousazadeh, Faezeh Babaieasl, Maysam Rezapour, Hamideh Hakimi

**Affiliations:** ^1^Department of Nursing, Amol School of Nursing and Midwifery, Mazandaran University of Medical Sciences, Sari, Iran; ^2^College of Nursing and Health Sciences, Department of Nursing Practice, Texas A & M University, Corpus Christi, Texas, USA; ^3^Department of Paramedicine, Amol School of Paramedical Sciences, Mazandaran University of Medical Sciences, Sari, Iran; ^4^Department of Nursing, Social Determinants of Health Research Center, Faculty of Nursing and Midwifery, Qazvin University of Medical Sciences, Qazvin, Iran

**Keywords:** compassionate care, multilevel modeling, nurses, resilience

## Abstract

**Background:** Compassionate care and resilience play crucial roles in enhancing care quality. Understanding compassionate care patterns in the nursing population and their relationship with resilience can improve knowledge of these concepts.

**Objective:** To investigate compassionate care patterns among Iranian nurses and their associations with resilience at individual and contextual levels.

**Method:** This cross-sectional study used Persian versions of the 28-item Compassionate Care Questionnaire (CCQ) and Connor–Davidson Resilience Scale (CD-RISC). 478 Iranian nurses from 19 wards across 16 hospitals in northern Iran participated between February and September 2023. Latent profile analysis (LPA) was performed on compassionate care dimensions. Multilevel logistic regression modeling explored associations between resilience dimensions and compassionate care profiles, adjusting for control variables at individual and contextual levels.

**Results:** LPA revealed two distinct compassionate care profiles: “Low Compassionate Care” (16.3%) and “High Compassionate Care” (83.7%). After adjusting for control variables, personal competence (OR = 1.09, 95% CI: 1.02–1.15), positive acceptance (OR = 1.23, 95% CI: 1.06–1.44), and spirituality (OR = 1.17, 95% CI: 1.01–1.38) were associated with higher odds of inclusion in the “High Compassionate Care” profile. The empty model showed that 6.5% of compassionate care patterns were explained at the ward level. After adding predictors at individual and ward levels, explained variances were reduced to 1.5% and < 0.01%, respectively.

**Conclusions and Implication:** This study offers insights into compassionate care among nurses, showing that few exhibited “Low Compassionate Care.” Higher resilience was positively linked to elevated compassionate care levels. Policymakers, managers, and educators can leverage these findings to enhance nursing management and support systems by prioritizing resilience-building strategies, ultimately improving patient care quality.

## 1. Introduction

Paul Gilbert, the founder of compassion-focused therapy (CFT), defines compassion as a sensitivity to the suffering of self and others, with a commitment to try to alleviate or prevent it [1]. This definition is widely accepted among researchers studying compassion and its components. Compassion is an integral part of modern nursing, described as a fundamental value in patient care [1–3]. It is central to the nursing profession's philosophical basis and context, with responding compassionately to patients' care needs considered a crucial standard [4–6]. The American Nursing Association's code of ethics explicitly calls for nurses to behave compassionately toward patients [6].

Providing compassionate care yields numerous benefits, including increased patient satisfaction, enhanced safety, reduced treatment costs, and improved nurses' job satisfaction and quality of life [4, 7]. It encourages patients to express their concerns more easily [8]. Conversely, a lack of compassion can lead to increased treatment costs, extended patient stays, more medical errors, and in extreme cases, even contribute to increased patient mortality [9–11].

The challenging nature of nursing work, including harsh work environments, caring for critically ill patients, and occupational hazards, can lead to stress, burnout, and decreased quality of care [12]. In such situations, resilience plays a crucial role. First proposed by Smith and Warner in psychology [13], resilience expresses an individual's capacity to adapt positively to difficult conditions and challenges. It is considered essential for nurses, as it increases job satisfaction, improves mental health, and ultimately enhances care delivery [14–16].

The concepts of compassion and resilience are inherently related. Compassion nurtures resilience, manifesting in both the response to and the outcome of situations involving suffering or distress [17]. As nurses are responsible for providing compassionate care [18], their mental and physical well-being directly impacts treatment goals and care quality [19]. Recognizing this, many healthcare institutions have invested in programs to increase resilience skills in healthcare workers [20–22]. However, studies across various departments have shown that a few nurses exhibit high levels of resilience [19, 23].

A few studies have investigated correlates of compassionate care among hospital nurses considering both person- and ward-level differences. This approach allows the examination of individual differences, ward-level differences, and contextual effects. While individual factors like gender, age, and education are frequently examined [24–26], resilience can be measured at both individual and contextual levels [27, 28]. Hospital wards significantly influence nurse behavior, and ward-level factors like perceptions of resilience have been linked to compassionate care [29].

Previous research has three main gaps: (1) Most studies were conducted in Western countries [22, 30], with limited research available from Eastern regions, indicating a need for a broader exploration of compassionate care and resilience across diverse healthcare settings [31–33], (2) few have examined patterns of compassionate care and their association with resilience dimensions, and (3) most focus on individual resilience perceptions, neglecting collective ward-level perceptions.

This study addresses these gaps by examining associations between individual perceptions of resilience subdomains (personal competence [PC], trust, positive acceptance, control [CO], and spiritual influence [SI]) and collective ward-level perceptions of total resilience with nurses' compassionate care patterns.

## 2. Research Design and Methods

This cross-sectional study involved 478 nurses from 17 wards across 16 public hospitals in X Province, northern Iran, conducted in 2023. Participants were bedside nurses with at least one year of work experience. After obtaining necessary approvals, researchers explained the study objectives and provided questionnaires to willing participants, assuring anonymity and voluntary participation.

### 2.1. Variables

The dependent variable was the pattern of compassionate care, identified through latent profile analysis (LPA). Control variables included demographic characteristics: age, sex (male/female), duty status (rotating/fixed), education level (BS/MSc and PhD), and marital status (married/single and other). Independent variables were individual perceptions of resilience subdomains: PC, trust, positive acceptance, CO, and SI. The ward-level factor was the collective perception of total resilience, calculated by averaging individual total resilience scores within each ward.

### 2.2. Instruments

Demographic characteristics form, and two standard questionnaires were used to collect data.

#### 2.2.1. Demographic Characteristics

The demographic variables included age, sex, duty status, education level, and marital status.

#### 2.2.2. Compassionate Care Questionnaire (CCQ)

The 28-item CCQ was used to assess compassionate care among nurses. This questionnaire includes dimensions of professional performance (PP), continuous follow-up (CF), patient-centered performance (PCP), and empathic communication. Response options are scored on a five-point Likert scale: “always” (5 points), “often” (4 points), “sometimes” (3 points), “rarely” (2 points), and “never” (1 point). Tehranineshat et al. found a Cronbach's alpha of 0.89 for the total score, with 0.83 for PP, 0.76 for CF, 0.73 for PCP, and 0.70 for empathic communication. The CCQ was also found to be moderately correlated (*r* = 0.68) with the Caring Behaviors Inventory (CBI-42) Wu, Larrabee, and Putman, suggesting convergent validity [33, 34]. In this study, Cronbach's alpha (*α*) was high for the total score (*α* = 0.96) and for the factors: P = 0.83, CF = 0.84, PCP = 0.82, and EC = 0.86.

#### 2.2.3. Connor–Davidson Resilience Scale (CD-RISC)

The CD-RISC is a 25-item self-report instrument rated on a 5-point scale (0–4), with higher scores reflecting greater resilience. The CD-RISC comprises five factors: PC, trust in individual instincts (TII), positive acceptance of change (PAC), CO, and SI [35]. In this study, Cronbach's alpha (*α*) was high for the total score (*α* = 0.95) and for the factors: PC = 0.87, TII = 0.79, PAC = 0.75, CO = 0.79, and SI = 0.71.

### 2.3. Data Analysis

Statistical analysis proceeded in two main phases: Descriptive statistics were reported for all variables.

LPA was conducted using Mplus 8.3 to identify subgroups of compassionate care patterns. LPA categorized nurses into homogeneous subgroups based on their perceptions of compassionate care dimensions: PP, CF, PCP, and empathic communication. To determine the optimal number of profiles, we examined multiple LPA models, starting with a two-profile model and gradually increasing the number of profiles. The best-fitting model was selected based on statistical criteria and interpretability of the profiles.

The statistical criteria were used the Akaike information criterion (AIC), Bayesian information criterion (BIC), sample size-adjusted Bayesian information criterion (ABIC), and the Lo–Mendell–Rubin likelihood ratio (LMR-LR) test, Vuong–Lo–Mendell–Rubin likelihood ratio test (VLMR). Lower BIC, AIC, and ABIC values indicate a better model fit. Model selection criteria included AIC, BIC, and ABIC (lower values indicating better fit), as well as LMR-LR and VLMR tests. These likelihood ratio tests compare the current model to one with one fewer class, aiding in determining the optimal number of profiles.

Model fit improvement was assessed using LMR and VLMR tests, with nonsignificant results indicating no improvement from adding another class. Entropy, ranging from 0 to 1, measured classification quality, with values closer to one indicating better classification.

Compassionate care dimensions and other variables were compared across patterns using independent *t*-tests with unequal variances and chi-square tests.

Given the nested structure of nurses within wards, multilevel logistic regression with random intercept was employed to explore individual and ward-level predictors of compassionate care patterns. This approach accounts for shared group variance and allows testing of between-person and between-ward dependencies. Three multilevel models were fit using Mplus 8.3.

We began with a null model (Model 1) to calculate the intraclass correlation coefficient, which indicated that 6.5% of the variance in compassionate care patterns was between wards. Despite this low correlation, it was necessary to account for between-ward dependencies due to a statistically significant random ward intercept variance. Comparison of models with and without random between-ward intercept showed that the latter had a significantly better fit (ΔLR = 7.75, Δdf = 1, *p* value = 0.0027).

Given these findings, we opted for a multilevel framework to adjust for group-level dependency. Model two incorporated individual-level variables (control variables and resilience subdomains), while Model 3 added a ward-level variable (aggregate total resilience). Model fit was assessed using reductions in −2 log likelihood, AIC, and BIC. Likelihood ratio tests were used to examine improvements in model fit across nested models.

### 2.4. Data Analysis

Statistical analysis proceeded in two main phases: (1) Descriptive statistics were reported for all variables and (2) LPA was conducted using Mplus 8.3 to identify subgroups of compassionate care patterns. LPA categorized nurses into homogeneous subgroups based on their perceptions of compassionate care dimensions: PP, CF, PCP, and empathic communication. To determine the optimal number of profiles, we examined multiple LPA models, starting with a two-profile model and gradually increasing the number of profiles. The best-fitting model was selected based on statistical criteria and interpretability of the profiles.

The statistical criteria used were the AIC, BIC, sample size-ABIC, LMR-LR test, and VLMR likelihood ratio test. Lower BIC, AIC, and ABIC values indicate better model fit. Model selection criteria included AIC, BIC, and ABIC (lower values indicating better fit), as well as LMR-LR and VLMR tests. These likelihood ratio tests compare the current model to one with one fewer class, aiding in determining the optimal number of profiles.

Model fit improvement was assessed using LMR and VLMR tests, with nonsignificant results indicating no improvement from adding another class. Entropy, ranging from 0 to 1, measured classification quality, with values closer to one indicating better classification. Compassionate care dimensions and other variables were compared across patterns using independent *t*-tests with unequal variances and chi-square tests.

Given the nested structure of nurses within wards, multilevel logistic regression with random intercept was employed to explore individual and ward-level predictors of compassionate care patterns. This approach accounts for shared group variance and allows testing of between-person and between-ward dependencies. Three multilevel models were fit using Mplus 8.3.

We began with a null model (Model 1) to calculate the intraclass correlation coefficient, which indicated that 6.5% of the variance in compassionate care patterns was between wards. Despite this low correlation, it was necessary to account for between-ward dependencies due to a statistically significant random ward intercept variance. Comparison of models with and without random between-ward intercept showed that the latter had a significantly better fit (ΔLR = 7.75, Δdf = 1, *p* value = 0.0027).

Given these findings, we opted for a multilevel framework to adjust for group-level dependency. Model 2 incorporated individual-level variables (control variables and resilience subdomains), while Model 3 added a ward-level variable (aggregate total resilience). Model fit was assessed using reductions in −2 log likelihood, AIC, and BIC. Likelihood ratio tests were used to examine improvements in model fit across nested models.

### 2.5. Ethical Considerations

All participants provided informed consent and were informed of their right to voluntary participation and withdrawal without consequences. Researchers maintained strict confidentiality of participant information. The study received ethical approval from the *X* Ethical Review Authority (Code: X.REC.1401.510).

## 3. Results

Table 1 presents descriptive statistics for nurses' perceptions of compassionate care dimensions, resilience dimensions, age, and total resilience at the ward level. The table also includes frequency and percentage distributions for gender, duty status, education level, and marital status.

**Figure 1:**
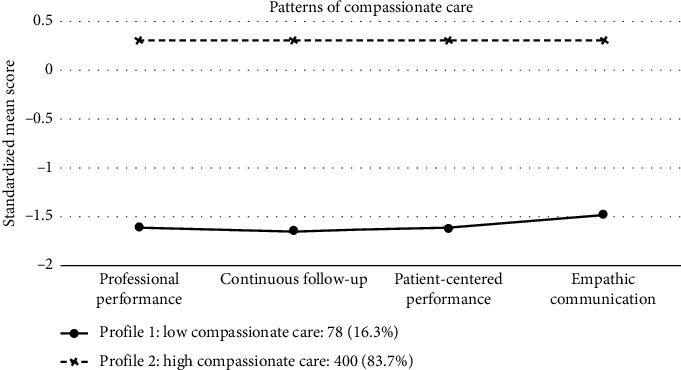
Standardized mean scores for compassion care dimensions across the two-profile solution.

LPA fit indices for compassionate care across 2 to 6 profiles are presented in Table 2. The two-profile model was identified as the best fit based on statistical criteria and interpretability. Profiles were labeled according to mean scores of compassionate care dimensions.  Figure 1 illustrates the standardized mean scores for these dimensions across the two profiles. Nurses in the “Low Compassionate Care” profile (16.3%) reported lower scores on all dimensions, while those in the “High Compassionate Care” profile (83.7%) reported higher scores on all dimensions.

Table 3 displays the distribution of compassionate care, resilience dimensions, and control variables across “Low Compassionate Care” and “High Compassionate Care” profiles. Significant differences were observed in means of resilience subdomains (PC, trust, positive acceptance, CO, and SI), compassionate care dimensions (PP, CF, PCP, and empathic communication), and age. Gender and marital status also showed significant differences between profiles, while duty status and education level did not.

Table 4 presents the associations between resilience dimensions and compassionate care patterns, adjusting for individual-level control variables (age, sex, duty status, education, and marital status) and ward-level collective total resilience using two-level logistic regression models. We started with a random intercept model (Model 1), then added individual-level variables (Model 2), and finally incorporated the ward-level variable (Model 3).

Model 1 showed that 6.5% of compassion care patterns were explained at the ward level, reducing to 1.5% and < 0.01% with the addition of individual-level and ward-level predictors, respectively. Models 2 and 3 revealed that increases in certain resilience dimensions were associated with higher odds of inclusion in the “High Compassionate Care” profile, after adjusting for control variables: PC (9% increase, OR = 1.09, 95% CI: 1.02, 1.15), positive acceptance (23% increase, OR = 1.23, 95% CI: 1.06, 1.44), and spirituality (17% increase, OR = 1.17, 95% CI: 1.01, 1.38). At the ward level, a one-unit increase in total resilience was associated with a 10% increase in the odds of inclusion in the “High Compassionate Care” profile (OR = 1.10, 95% CI: 1.02, 1.19).

Among control variables, gender and marital status were significant predictors. Males had lower odds of inclusion in the “High Compassionate Care” profile compared to females (OR = 0.28, 95% CI: 0.18, 0.43), while married nurses had higher odds compared to single nurses (OR = 1.96, 95% CI: 1.17, 3.29). Nested models were evaluated using log-likelihood ratio tests, AIC, BIC, and sample size-ABIC. Model 2 significantly improved fit compared to Model 1 (ΔLR = 104.42, Δdf = 10, *p* value < 0.001), but Model 3 did not significantly improve fit compared to Model 2 (ΔLR = 3.31, Δdf = 1, *p* value = 0.061).

## 4. Discussion

This study explored compassionate care patterns among Iranian nurses and their associations with resilience at individual and contextual levels. Two distinct profiles emerged: “High Compassionate Care” (83.7%) and “Low Compassionate Care” (16.3%), aligning with previous research on varying levels of compassionate care among nurses [36–38].

Higher levels of PC, PA, and spirituality (resilience dimensions) were associated with an increased likelihood of inclusion in the “High Compassionate Care” profile. These findings support previous studies linking resilience to compassionate care [39]. Resilient nurses are better equipped to manage emotional demands and maintain compassionate care practices [40].

The study also emphasized the importance of contextual factors by examining collective resilience at the ward level. Higher collective resilience was associated with an increased likelihood of inclusion in the “High Compassionate Care” profile. This aligns with the theory that workplace environments shape individual behaviors and attitudes, suggesting that resilient wards foster supportive environments conducive to compassionate care practices [41–43].

Results indicated that age was related to compassionate care, with married individuals and women demonstrating higher levels compared to men. This finding corroborates Lee and Seomun study, which reported that compassion in nurses increases with age, with 50-year-olds showing higher levels than their 20- and 30-year-old counterparts [36].

Park and Park's Korean study found that compassion competence increased with age [37] (35), though other research showed no significant relationship among age, gender, marriage, and compassionate care [24, 38, 44] (24, 36, 42). Compassionate care theory emphasizes motivation, sensitivity, empathy, sympathy, distress tolerance, and nonjudgment as key characteristics [45] (43). Compassion competence predicts nurses' caring behavior [38] (36), and Ghafari, Khani, and Mahmmodi demonstrated that compassion for others mediates between moral identity and nurses' compassionate care [46] (44). Studies indicate higher empathy, sensitivity, and attention to others in married women compared to single men [46, 47], with more frequent caring behaviors reported in female nurses [48]. This suggests higher compassionate care in women and married individuals. Additionally, nurses' moral sensitivity, which is linked to patient interest and understanding, increases with age [49], potentially explaining age-related increases in compassionate care.

This study identified two compassionate care profiles: high and low compassion, with consistent patterns across all domains within each profile. Most nurses fell into the “High Compassionate Care” profile, aligning with Park and Park findings of high compassion competence in oncology nurses [37]. However, Iranian studies have reported average or low compassion levels in nurses [24, 44]. Low compassion negatively impacts nursing care quality. Dehghani et al. found that decreased compassionate care leads to increased missed care [49], while Duarte, Pinto-Gouveia, and Cruz demonstrated that low nurse compassion reduces patient satisfaction [50].

The study found that increased PC, PA, and spirituality (resilience dimensions) are associated with a higher likelihood of exhibiting “High Compassionate Care” after adjusting for control variables. While no research has directly examined the relationship between compassionate care and resilience, related studies offer insights. Klimentidou et al. found that higher compassion in military nursing officers correlated with better quality of life, higher care satisfaction, and less burnout [51]. Altruism, sincerity, and empathy can enhance nurses' quality of life [52, 53]. Lee et al. reported a positive association between quality of life and compassion levels [36]. Studies on resilience and quality of life indicate that more resilient nurses have better life quality [40, 52, 54]. Park et al. found that oncology nurses with higher compassion competence respond more effectively to workplace stress and show higher resilience [37]. High compassionate competence is crucial for providing compassionate care [55], and resilience in stressful conditions improves caring behavior [44].

These findings have practical implications for healthcare organizations. Interventions to enhance individual resilience, such as mindfulness training and stress management programs, could improve compassionate care among nurses [39, 43]. Additionally, fostering a resilient ward culture through effective leadership and team-building may create an environment conducive to compassionate care practices [39].

## 5. Limitations

This study has several limitations. Its cross-sectional design precludes causal inferences, necessitating future longitudinal or experimental studies to establish causal relationships between resilience and compassionate care patterns. Sample size constraints prevented the inclusion of interaction terms in regression models. The study's generalizability is limited due to its focus on governmental hospitals in one Iranian province. Reliance on self-report measures may introduce response biases, suggesting the need for objective measures in future research. Lastly, potential mediating or moderating variables were not considered, presenting an opportunity for future studies to explore these factors and better understand the underlying mechanisms of the relationship between resilience and compassionate care patterns.

## 6. Conclusions

This study offers valuable insights into compassionate care patterns among nurses and their links to resilience at individual and ward levels. Findings show that 83.7% of nurses demonstrate high levels of compassionate care, significantly influenced by personal and collective ward resilience. The implications for nursing management and healthcare organizations are substantial, highlighting the need to prioritize resilience-building strategies. By implementing training programs, mentorship initiatives, and supportive work environments, healthcare organizations can empower nurses to sustain compassionate care even in challenging situations.

Future research could explore the long-term effects of resilience-enhancing interventions on compassionate care, cross-cultural factors affecting this relationship, and targeted studies on specific resilience subdomains. Focusing on resilience can help create a more compassionate and effective nursing workforce, ultimately improving patient care and organizational outcomes. Integrating resilience-focused strategies into nursing education and policies is essential for developing a sustainable workforce dedicated to high-quality patient care.

## Figures and Tables

**Table 1:** Descriptive statistics for all variables in this study (*n* = 478).

	Mean ± SD	Min, max
Compassionate care dimensions		
Professional performance	41.39 ± 4.59	[9, 45]
Continuous follow-up	26.23 ± 3.86	[6, 30]
Patient-centered performance	29.45 ± 4.64	[7, 35]
Empathic communication	26.31 ± 3.62	[6, 30]
Individual-level variables		
Gender	*n* (%)	
Female	367 (76.78%)	
Male	111 (23.22%)	
Age	34.91 ± 8.10	
Duty status	*n* (%)	
In changing	403 (84.31%)	
Fix	75 (15.69%)	
Education	*n* (%)	
Bs	428 (89.54%)	
MSc and PhD	49 (10.25%)	
Marital status	*n* (%)	
Single and other	140 (29.29%)	
Married	337 (70.50%)	
Resilience dimensions	Mean ± SD	Min, max
Personal competence	31.51 ± 5.65	[8, 40]
Confidence	26.43 ± 4.58	[7, 35]
Positive acceptance	19.84 ± 3.07	[5, 25]
Self-control	11.77 ± 2.26	[3, 15]
Spirituality	8.06 ± 1.72	[2, 10]
Ward-level variable		
Resilience	99.51 ± 32.69	[88, 115]

**Table 2:** Latent profile analysis fit indices for compassionate care patterns.

	Log likelihood	AIC	BIC	aBIC	LMR-LRT	VLMR-LRT	Entropy	Sample size
2 Profiles	−4967.4	9960.9	10015.1	9973.9	901.8⁣^∗^	931.1⁣^∗^	0.94	78 (16.3%)/400 (83.7%)
3 Profiles	−4756.9	9549.9	9624.9	9567.8	407.8	421	0.886	42 (8.8%)/158 (33.0%)/278 (58.2%)
4 Profiles	−4672.9	9391.9	9487.8	9414.8	162.6⁣^∗∗∗^	167.9⁣^∗∗∗^	0.918	1 (< 0.01%)/273 (5.71%)/43 (9.0%)/161 (33.6%)
5 Profiles	−4610.6	9277.3	9394.1	9305.2	124.6⁣^∗^	122.4⁣^∗^	0.887	1 (< 0.01%)/163 (34.1%)/27 (5.6%)/231 (4.83%)/56 (11.7%)
6 Profiles	−4576.6	9219.3	9356.9	9252.2	66.7	67.9	0.881	1 (< 0.01%)/20 (4.2%)/150 (31.4%)/25 (5.2%)/75 (15.7%)/207 (43.3%)

*Note:* LMR, Lo–Mendell–Rubin likelihood ratio test; VLMR, Vuong–Lo–Mendell–Rubin likelihood ratio test.

Abbreviations: AIC, Akaike information criterion; BIC, Bayesian information criterion; SSABIC, sample size-adjusted Bayesian information criterion.

**Table 3:** The distribution of compassionate care, resilience dimensions, and control variables across over the compassionate care patterns.

	Low compassionate care	High compassionate care	Test statistics	*p* value
	78 (16.32%)	400 (83.68%)		
Compassionate care dimensions				
Professional performance	33.98 ± 5.56	42.83 ± 2.52	13.76^A^	< 0.001
Continuous follow-up	19.82 ± 3.20	27.47 ± 2.49	19.96^A^	< 0.001
Patient-centered performance	21.88 ± 3.89	30.92 ± 3.07	19.37^A^	< 0.001
Empathic communication	20.88 ± 3.45	27.37 ± 2.54	15.78^A^	< 0.001

Individual-level variables				
Gender			21.63^B^	< 0.001
Female	44 (56.41%)	323 (80.75%)		
Male	34 (43.59%)	77 (19.25%)		
Age	31.71 ± 7.15	35.54 ± 8.13	4.23^A^	< 0.001
Duty status			2.08^B^	0.149
In changing	70 (89.74%)	333 (83.25%)		
Fix	8 (10.26%)	67 (16.75%)		
Education			0.85^B^	0.653
Bs	68 (87.18%)	360 (90.0%)		
MSc and PhD	10 (12.82%)	39 (9.75%)		
Marital status			14.92^B^	0.001
Single and other	37 (47.44%)	103 (25.75%)		
Married	41 (52.56%)	296 (74.0%)		
Resilience dimensions				
Personal competence	27.07 ± 5.56	32.37 ± 5.25	7.75^A^	< 0.001
Confidence	23.01 ± 4.33	27.10 ± 4.32	7.64^A^	< 0.001
Positive acceptance	17.25 ± 3.55	20.34 ± 2.75	7.63^A^	< 0.001
Self-control	10.77 ± 2.37	12.05 ± 2.13	5.95^A^	< 0.001
Spirituality	6.74 ± 1.89	8.32 ± 1.55	6.91^A^	< 0.001

Ward-level variable				
Resilience	84.48 ± 35.26	100.24 ± 38.22	9.02^A^	< 0.001

^A^Two-sample *t* test with unequal variances.

^B^Chi-square test.

**Table 4:** Estimates of fixed and random effects from multilevel logistic regression models for compassion care patterns.

	Model 1	Model 2	Model 3
	OR (95% CI)	OR (95% CI)	OR (95% CI)
Individual-level variables			
Gender (ref: female)		0.30 (0.19, 0.43)⁣^∗∗∗^	0.28 (0.18, 0.43)⁣^∗∗∗^
Age		1.03 (0.99, 1.07)	1.03 (0.99, 1.08)
Duty status		0.67 (0.25, 1.52)	0.57 (0.22, 1.49)
Education		0.84 (0.34, 1.81)	0.84 (0.35, 2.05)
Marital status		1.94 (1.15, 3.02)⁣^∗^	1.96 (1.17, 3.29)⁣^∗^
Personal competence		1.09 (1.02, 1.16)⁣^∗^	1.09 (1.02, 1.15)⁣^∗∗^
Confidence		1.07 (0.98, 1.14)	1.07 (0.99, 1.16)
Positive acceptance		1.23 (1.05, 1.41)⁣^∗^	1.23 (1.06, 1.44)⁣^∗^
Self-control		0.86 (0.70, 1.01)	0.87 (0.72, 1.04)
Spirituality		1.19 (1.01, 1.38)⁣^∗^	1.17 (1.01, 1.38)⁣^∗^
Ward-level variable			
Resilience			1.10 (1.02, 1.19)⁣^∗^
Variance of random effects (SE)	0.23 (0.14)	0.05 (0.05)	< 0.01 (< 0.01)
Log likelihood	−208.78	−156.566	−154.913
Akaike information criterion (AIC)	421.5	337.132	335.827
Bayesian information criterion (BIC)	429.9	387.117	389.977
Sample size-adjusted BIC	423.5	349.03	348.717

*Note:* Model 1 to Model 2: ΔLR = 104.42, Δdf = 10, *p* value < 0.001; Model 2 to Model 3: ΔLR = 3.31, Δdf = 1, *p* value = 0.061.

⁣^∗^*p* value < 0.05.

⁣^∗∗^*p* value < 0.01.

⁣^∗∗∗^*p* value < 0.001.

## Data Availability

The data that support the findings of this study are available from the corresponding author, but restrictions apply to the availability of these data, which were used under license for the current study, and so are not publicly available.
